# Biological Sex and Sex Hormone Impacts on Deficits in Episodic-Like Memory in a Rat Model of Early, Pre-motor Stages of Parkinson's Disease

**DOI:** 10.3389/fneur.2020.00942

**Published:** 2020-09-17

**Authors:** Meagan R. Conner, Doyeon Jang, Brenda J. Anderson, Mary F. Kritzer

**Affiliations:** ^1^Graduate Program in Neuroscience, Stony Brook University, Stony Brook, NY, United States; ^2^Department of Neurobiology and Behavior, Stony Brook University, Stony Brook, NY, United States; ^3^Department of Psychology, Stony Brook University, Stony Brook, NY, United States

**Keywords:** 6-OHDA, androgen, estrogen, dopamine, mild cognitive impairment, neostriatum

## Abstract

Episodic memory deficits are among the earliest appearing and most commonly occurring examples of cognitive impairment in Parkinson's disease (PD). These enduring features can also predict a clinical course of rapid motor decline, significant cognitive deterioration, and the development of PD-related dementia. The lack of effective means to treat these deficits underscores the need to better understand their neurobiological bases. The prominent sex differences that characterize episodic memory in health, aging and in schizophrenia and Alzheimer's disease suggest that neuroendocrine factors may also influence episodic memory dysfunction in PD. However, while sex differences have been well-documented for many facets of PD, sex differences in, and sex hormone influences on associated episodic memory impairments have been less extensively studied and have never been examined in preclinical PD models. Accordingly, we paired bilateral neostriatal 6-hydroxydopamine (6-OHDA) lesions with behavioral testing using the What-Where-When Episodic-Like Memory (ELM) Task in adult rats to first determine whether episodic-like memory is impaired in this model. We further compared outcomes in gonadally intact female and male subjects, and in male rats that had undergone gonadectomy—with and without hormone replacement, to determine whether biological sex and/or sex hormones influenced the expression of dopamine lesioned-induced memory deficits. These studies showed that 6-OHDA lesions profoundly impaired recall for all memory domains in male and female rats. They also showed that in males, circulating gonadal hormones powerfully modulated the negative impacts of 6-OHDA lesions on What, Where, and When discriminations in domain-specific ways. Specifically, the absence of androgens was shown to fully attenuate 6-OHDA lesion-induced deficits in ELM for “Where” and to partially protect against lesion-induced deficits in ELM for “What.” In sum, these findings show that 6-OHDA lesions in rats recapitulate the vulnerability of episodic memory seen in early PD. Together with similar evidence recently obtained for spatial working memory, the present findings also showed that diminished androgen levels provide powerful, highly selective protections against the harmful effects that 6-OHDA lesions have on memory functions in male rats.

## Introduction

Parkinson's Disease (PD) is a complex neurodegenerative disorder that is characterized by motor signs such as bradykinesia and non-motor symptoms that include deficits in sensory processing, sleep disturbance, and cognitive impairment (1–4). Parkinson's disease is also characterized by sex differences in many of its features (5–9). For example, the incidence and prevalence of PD are both higher in males than in females (10–13). Male PD patients also tend to experience earlier disease onset (14, 15) and more rapid declines in motor function (12). By understanding how gonadal hormones influence these and other disease processes, critical discoveries that could lead to improved, perhaps sex-specific ways to more effectively treat them. This may be especially important for the cognitive impairments associated with PD. These include deficits in executive, mnemonic and/or visuospatial function (4, 16–20) and are present in up to 40% of patients at or before the onset of motor signs (20, 21). Because these signs are also largely resistant to (19, 20, 22) or exacerbated by (23–25) available therapeutics, the also have a cumulative prevalence of more than 70% over the course of illness (20, 26). Moreover, these enduring, progressively worsening facets of disease (17–20) are frequently described by patients and caregivers as significantly disabling and negatively interfering with activities and quality of daily life (27, 28). This brings into sharp focus the need to better understand the factors that mediate and/or modulate the vulnerability of these complex processes in PD. As described below, these factors include biological sex and sex hormones, which are examined here in specific contexts of episodic memory deficits in PD.

Memory disturbances are among the earliest and most frequently reported cognitive deficits in PD (4, 16, 18, 29). For example, roughly 20% of newly diagnosed PD patients present with memory complaints and/or have demonstrable memory impairments at the time of clinical diagnosis (30). One domain of memory that is especially at risk, however, is episodic memory, i.e., the integrated recall of information about the time, place and nature of previously experienced events (31). Thus, deficits in episodic memory are reported among PD patients as often, or even more frequently than executive dysfunction (21, 32, 33). Further, the presence of episodic memory deficits has been shown to correlate with or predict certain clinical features including accelerated rates of motor and cognitive decline (34, 35), and higher risk for developing PD-related dementia (26, 36, 37)– a major cause of hospitalization, institutionalization and death among PD patients (38–41). These characteristics suggest that resolving the factors that render episodic memory vulnerable in PD may be uniquely important in identifying biomarkers and/or biological targets with predictive, preventive, and/or therapeutic value. Accordingly, imaging and autopsy studies in PD patients have been used to probe for brain changes that predict and/or correlate with the severity of episodic memory impairment. These studies have identified thinning or decreased volume in hippocampus and in medial temporal and frontal lobe cortices (42–44) as well as increased total volumes of white matter hyperintense lesions in the frontal and temporal lobes (45) as promising candidates. Although less is known about the physiological processes that render episodic memory vulnerable in PD, there are reasons to suspect that biological sex and/or sex hormones play important roles. First, episodic memory is characterized by lifelong sex differences in healthy humans (46–49). Further, sex differences also mark the incidence and severity of episodic memory deficits seen during cognitive aging (47, 50, 51) in schizophrenia (52, 53) and in Alzheimer's disease (54–57). Finally, although less studied than motor features, there is clear evidence for sex differences in cognitive impairment in PD (5, 6, 58, 59) including data showing that episodic memory deficits are more common and worsen more rapidly in males (60) and respond better to multimodal exercise interventions in females (61, 62). To date, however, there have been no studies that take advantage of preclinical animal models to more precisely determine whether and how biological sex and sex hormones influence episodic memory in PD. We recently validated the use of the What Where When Episodic Like Memory task (63) in adult male and female rats for modeling human sex differences in episodic memory function, and in adult male rats that were gonadectomized or gonadectomized and supplemented with testosterone propionate or estradiol for identifying hormone impacts on this memory form (64). Here we used this same task and these same animal groups, but added sham and partial, bilateral neostriatal 6-hydroxydomapine (6-OHDA) lesions to probe for sex differences in and sex hormone impacts on episodic-like memory function in a rodent model of early, premotor stages of PD (65, 66).

## Materials and Methods

### Animal Subjects

A total of 22 female and 55 male Sprague-Dawley rats were used (Taconic Farms, Germantown, New York, USA). All subjects were between 2 and 2.5 months of age at the beginning of the experiment, and were 3–3.5 months old at the time of behavioral testing. All rats received bilateral neostriatal injections of either 6-hydroxydopamine (6-OHDA) or vehicle (below). The females were gonadally intact. Among the males, 22 were gonadally intact, 11 were gonadectomized (GDX), 11 were GDX and supplemented with testosterone propionate (GDX+TP), and 11 were GDX and supplemented with 17β-estradiol (GDX+E). Rats were pair-housed by sex in standard-sized tub cages (Lab Products, Inc., Seaford, DE, USA) under a 12-h non-reversed light-dark cycle with food (Purina PMI Lab Diet: ProLab RMH 3000) and water available ad libitum. The cages and water bottles were made from bisphenol–free plastic (Zyfone) and ground corncob bedding (Bed O' Cobs, The Anderson Inc., Maumee, Ohio, USA) was used. All procedures involving rats were approved by the Institutional Animal Care and Use Committee at Stony Brook University and were performed in accordance with the U.S. Public Health Service Guide for Care and Use of Laboratory Animals to minimize their discomfort.

### Surgeries

Surgical procedures were performed under aseptic conditions using inhalation of isoflurane (1% in oxygen) as anesthesia and subcutaneous injection of buprenorphine (0.03 mg/kg) or ketorolac (3 mg/kg) for post-operative analgesia.

#### Gonadectomy

For this procedure, a midline incision was made to the scrotum, the vas deferens were bilaterally ligated with silk suture, and the testes were removed. For hormone-supplemented rats, pellets (Innovative Research of America, Sarasota, Florida) were implanted at the surgical site; the pellets used were designed to release either 25 pg of 17β -estradiol per milliliter of blood per day or 3–4 ng of testosterone propionate per milliliter of blood per day. These pellets have been shown in previous investigations to maintain plasma estradiol and testosterone levels within physiological ranges (67–69). Two weeks after this procedure, rats received 6-OHDA lesions (below).

#### 6-OHDA and SHAM Lesions

Thirty minutes prior to surgery, all rats were injected with desipramine hydrochloride (20 mg/kg, intraperitoneal). Next, rats were anesthetized (isoflurane) and placed in a stereotaxic frame. A midline incision was made to expose the skull surface and burr holes were drilled bilaterally at coordinates targeting the middle one/third of the left and right rostral caudate nucleus (AP: +0.5 mm, ML: ±3.0 mm, relative to Bregma). A glass micropipette containing either 6-hydroxydopamine (6-OHDA) (6 μg/μL) dissolved in ascorbic saline (de-ionized water containing 0.9% NaCl and 0.1% ascorbic acid), or ascorbic saline alone (SHAM), was lowered 5.8 mm below the dura. As the pipette was withdrawn, solution was intermittently ejected at roughly 2–5 min intervals (Nanoject, Drummond Scientific, Broomall, PA, infusion rate ~0.15 μl/min) at 11 evenly-spaced dorsoventral depths located between 5.8 and 3.8 mm below the dura; the total injected volume was 2 μl/hemisphere. Following the last injection, the micropipette was kept in place for an additional 10 min before being slowly withdrawn. Two weeks later, rats began a regimen of handling, habituation, and behavioral testing that culminated to testing on the What-Where-When (WWWhen) ELM task 10 days after that (24 days after lesion surgery).

### Behavioral Testing

Testing took place during rats' subjective night (lights on), between the hours of 9:30 am. and 3:00 pm. By conducting studies during these earlier hours of rats' subjective nights, testing times within and across female subjects avoided the potential for overlap with the precipitous decreases in estrogen, the rapid increases in follicle stimulating hormone, and the rapid increases and subsequent sharp declines in progesterone and luteinizing hormone that begin toward the end of rats' subjective nights and continue into the subjective day (70). Testing was conducted in a core facility consisting of a central holding room and 5 adjacent, sound attenuated testing rooms. The testing arena used was an open circular platform that was 0.61 m in diameter and located 0.91 m above the floor (padded) near the center of a 3 m by 3 m testing room with fixed, high contrast visual cues on three walls. The platform surface was covered by black vinyl to provide grip and allow the platform to be wiped clean with 70% ethanol between trials. A webcam (LogiTech) was suspended two feet above the arena to record trials.

Prior to WWWhen testing, rats completed testing on 2 or 3 unrelated, non-incentivized, pseudorandom presented single-trial paradigms (Home Base Formation and Novel Object Preference or Object-in-Place Preference Testing). Each task used a different testing arena and different objects (where applicable), but the same holding and testing rooms, and the same cylindrical start box used for WWWhen testing. After a 3–5 day break, rats received an additional habituation session during which they were allowed to freely explore the WWWhen testing platform with no objects present for 10 min. Testing on the WWWhen task took place the following day.

**Figure 1:**
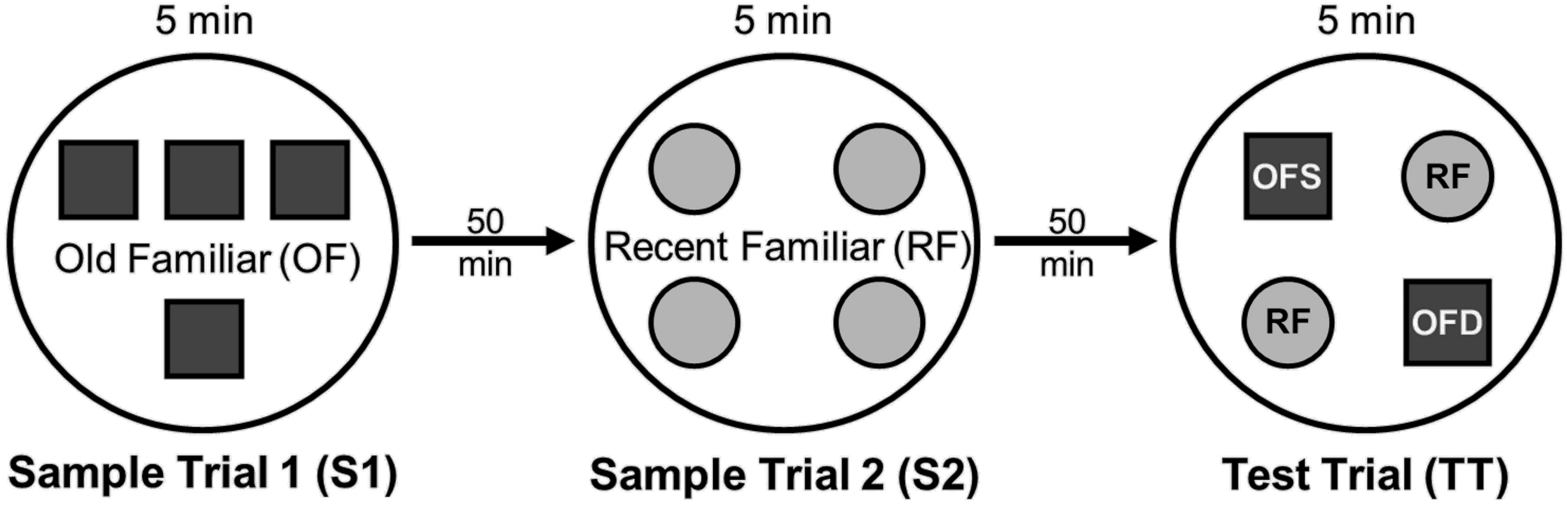
Schematic of the experimental protocol for the What-Where-When Episodic-Like Memory Task (63). Rats explore two sets of novel quadruplicate objects during a first and second sample trial (S1 and S2, respectively). This is followed by a test trial (TT) where two Recent Familiar (RF) objects from S2 are presented in their original positions and two Old Familiar (OF) objects from S1 are present, with one placed in its original position [Old Familiar Stationary (OFS)] and one that has been displaced from its original location [Old Familiar Displaced (OFD)]. Each trial is 5 min in duration and separated by an inter-trial interval of 50 min.

On testing day, rats were given two 5 min sample trials (S1, S2) and one 5 min test trial (TT); all trials were separated by 50 min inter-trial intervals that rats spent in home cages in the central holding room. Trials began by placing rats in the opaque start cylinder (0.2 m across, 0.3 m high) at the center of the arena. After a 10 s delay, the cylinder was lifted away and rats were allowed to freely explore. During the sample trials (S1, S2), rats were presented with one of two quadruplicate sets of novel objects that were similar in overall size, were made of the same material (plastic or glass), but differed in shape, color and/or surface texture. The object sets used for S1 vs. S2 trials were pseudo-randomly assigned to subjects within all groups. During the S1 trial, objects were arranged in a triangular array and for the S2 trial they were placed to form the corners of a square. During the test trial (TT), two objects from S2 (recent familiar, RF) and two objects from S1 (old familiar, OF) were presented in square S2 configuration. Accordingly, one of the S1 objects appeared in its original location (old familiar stationary, OFS) and was presented in a new location (old familiar displaced, OFD). Both recent familiar objects occupied original (stationary) positions (see Figure 1).

### Health and Hormone Status

The weights and general health of all subjects were tracked before and after gonadectomy and/or 6-OHDA or SHAM lesion surgery. In all subjects, body mass progressively increased and there were no signs of dehydration. The estrous cycles of female rats were also tracked using vaginal lavage. Cytological samples were collected via saline flush on the day of lesion or sham lesion surgery, on the day of WWWhen testing, and every 1–3 days in between. On testing day, samples were collected prior to animals' acclimation to the central holding room in the testing suite. For males, the effectiveness of hormone manipulations was assessed post-mortem via dissection and weighing of the androgen-sensitive bulbospongiosis muscles (BSM).

### Euthanasia

Following behavioral testing, rats were euthanized by transcardial perfusion. First, rats were lightly anesthetized using inhalation of isoflurane (1% in oxygen) and were then injected intraperitoneally with a ketamine (150 mg/kg), xylazine (15 mg/kg) mixture to induce deep anesthesia. After verifying the absence of deep reflexes, rats were perfused, first with phosphate buffered saline (PBS, ~100 mls), and then with 4% paraformaldehyde in 0.1M PB, pH 6.5 (flow rate 30 ml/min) for 5 min followed by 4% paraformaldehyde in 0.1M borate buffer, pH 9.5 (flow rate 35 ml/min) for 15 min.

#### Tissue Processing and Histology

Immediately after perfusion, the BSM complex in male subjects were dissected and weighed. However, for 4 MALE SHAM and 4 MALE 6-OHDA rats, BSM weights were not recovered. The brains were also removed from all subjects. These were post-fixed in 4% paraformaldehyde in 0.1M borate buffer for 24 h (4°C) and were then cryoprotected by immersion in 0.1M phosphate buffer (PB) containing 30% sucrose (2–5 days, 4°C). Next, the brains were rapidly frozen in powdered dry ice and serially sectioned in the coronal plane on a freezing microtome (40 μm). A one-in-six series of sections spanning the rostrocaudal extent of the caudate nucleus was processed for immunohistochemistry using antibodies against the dopamine-synthesizing enzyme, tyrosine hydroxylase (TH). Briefly, sections were rinsed in 0.1M PB, incubated in 0.1M PB containing 1% H_2_O_2_ (30 min), and were then rinsed again in 0.1M PB prior to an antigen retrieval step involving a 20 min incubation in sodium citrate buffer, pH 8.5 at 80°C. After rinses in tris buffered saline (TBS), pH 7.4, the sections were placed in TBS, pH 7.4 containing 10% normal swine serum (NSS) for 2 h, and then in primary antisera (anti-TH monoclonal antibody; Chemicon International Inc, Temecula, CA; MAB318, diluted 1:1000 in TBS containing 1% NSS) for 4 days (4°C). Following further rinses in TBS, pH 7.4, the sections were incubated overnight in biotinylated secondary antibody (Vector Laboratories, Burlingame, CA, USA, diluted 1:100 in TBS containing 1% NSS, 4°C). Next, sections were rinsed in TBS, pH 7.4 and incubated in avidin-complexed horseradish peroxidase (ABC, Vector Laboratories, 5 h, room temperature). Finally, the sections were rinsed first in TBS pH 7.4 then in TB, pH 7.6 before being reacted using 3–3′ diaminobenzidine as chromagen and 1% H_2_O_2_ as catalyst. The immunoreacted sections were then slide mounted and sealed under coverslips using Permount (Electron Microscopy Science, Hartfield PA, USA).

### Data Analyses

#### Evaluation of Estrous Cycle

Samples from vaginal lavage (saline) were cytologically evaluated using low power light microscopy and differential interference contrast illumination. Females were determined to be in estrus when samples had an abundance of cornified, anucleated epithelial cells; in diestrus when samples showed a predominance of leukocytes; and in proestrus when samples were largely comprised of round, nucleated epithelial cells (70).

#### Efficacy of GDX and Hormone Replacement

The extents to which GDX and hormone replacement modulated circulating hormone levels were assessed by comparing weights of the androgen-sensitive bulbospongiosis muscles across male groups.

#### Evaluations of 6-OHDA Lesions

A Zeiss Axioskop outfitted with an Infinity 3 Lumenera digital camera was used to collect low-power brightfield light microscopic images of TH-immunoreacted sections. These images were imported into ImageJ (Open Source, 1.52a) and separate, calibrated outlines were drawn around the entire caudate nucleus (excluding nucleus accumbens) and around the lesioned zones, defined as areas within the caudate where TH-immunostaining fell to background levels. The areas subtended by both sets of outlines were used along with section thickness and numbers of sections per case to calculate total caudate and total lesion volumes on per hemisphere, per subject bases. Lesion symmetries were defined as the ratio of the larger compared to the smaller lesion volume, regardless of whether it was in the left or right hemisphere.

#### Behavioral Analyses

Behavioral scoring was completed by trained observers who were blind to animal condition using event capture (Behavioral Observation Research Interactive Software—BORIS, 7.0.4, Open Source) and tracking software (Tracker 4.62, Open Source). For all trials, scored behaviors were defined and measured as follows:

**Latency to Investigate Objects:** time (seconds) between the start of the trial (removal of the start box) and the first investigatory contact with an object.
**Grooming:** time (seconds) spent licking or preening any part of the body.
**Rearing:** time (seconds) spent making upward/vertical motion either with forepaws in contact with an object (without vibrissae or snout in contact with the object) or while free-standing.
**Ledge Investigation:** time (seconds) spent at and actively investigating the ledge/edge of the arena or looking out into the surrounding testing room environment.
**Ambulation:** time (seconds) spent engaged in forward motion, calculated from changes in position magnitudes measured in digitized tracks of rats' movement across the arena surface on per frame bases (Tracker 4.62).
**Stationary:** time (seconds) spent sitting in one location away from the arena edge, and not engaging in grooming or object exploration behaviors.
**Object Exploration:** time (seconds) spent in physical contact with objects, actively using vibrissae or snout to investigate, with or without rearing.
**Discrimination Indices (DI):** calculated during Test Trials based on time (seconds) spent exploring objects designated as recent familiar (RF), old familiar (OF), old familiar stationary (OFS), or old familiar displaced (OFD):

$$\begin{array}{l} \;WHAT\;DI=\left[average\;OF-average\;RF\right]\;÷\;\left[average\;OF+average\;RF\right]\\ WHERE\;DI=\left[OFD-OFS\right]\;÷\;\left[OFD+OFS\right]\\ \;WHEN\;DI=\left[OFD-average\;RF\right]\;÷\;\left[OFD+average\;RF\right] \end{array}$$

### Statistics

Statistical comparisons were used to evaluate behavioral endpoints for (1) sex differences [i.e., contrasts of gonadally intact sham-operated females (FEM SHAM) and males (MALE SHAM)], (2) sex differences in sensitivity to 6-OHDA lesions [i.e., contrasts of FEM SHAM and MALE SHAM with gonadally intact 6-OHDA lesioned females (FEM 6-OHDA) and males (MALE 6-OHDA)], and (3) sex hormone modulation of sensitivity to 6-OHDA lesions in males [i.e., contrasts of MALE SHAM and MALE 6-OHDA with the 6-OHDA lesioned GDX and hormone replacement cohorts (GDX 6-OHDA, GDX+TP 6-OHDA, GDX+E 6-OHDA)]. Due to small and uneven sample sizes, no attempts were made to statistically assess effects of estrous cycle stage among females.

All statistical analyses were performed using IBM SPSS, Version 25 (SPSS, Inc., Chicago, IL, USA). Data sets were first evaluated using descriptive statistics and tests for homogeneity of variance (Levine's *F*-test). From there, one-way analyses of variance (ANOVA) were used to compare BSM weights, lesion size, and lesion symmetry across groups. Repeated-measures ANOVAs were used to compare: all measures of object exploration, including DIs across groups; all measures of Non-Object Exploration (Other) behaviors within and across groups; and to evaluate within-groups differences in Other Behaviors across trials. For these comparisons, Mauchly's test for sphericity of the covariance matrix was applied and degrees of freedom were corrected as necessary using the Huyhn-Feldt epsilon. Allowed *post-hoc* tests used the Fisher's Protected Least Significant Difference (PLSD); for comparisons of two groups, a *p* < 0.05 level was accepted as significant; for comparisons of multiple groups, significance was determined using a Bonferroni corrected alpha (*p* < 0.0491 for comparisons of the 4 gonadally intact female and male groups and *p* < 0.0489 for comparisons of the 5 male groups). Robustness of object discriminations was assessed within groups for Test Trials using one-sample *t*-tests to identify DIs as significantly different from zero. Lastly, regression analyses were used within groups to evaluate 6-OHDA-induced lesion size as a function of BSM weight and to evaluate task performance metrics as functions of 6-OHDA lesions.

## Results

### Estrous Cycle in Females and Effectiveness of Hormone Manipulations in Males

Vaginal lavage samples obtained from female subjects (FEM SHAM, FEM 6-OHDA) showed that on the day of SHAM or 6-OHDA lesion surgery, 3 females from the FEM SHAM group were in estrus, 6 were in diestrus, and 2 were in proestrus, whereas among the FEM 6-OHDA group, 3 females were in estrus, 5 were in diestrus, and 2 were in proestrus at the time surgery was performed (Table 1). All subjects were also found to have resumed and retained a regular 4-day estrous cycle after surgery. On the day of WWWhen testing, 5 females from the FEM SHAM group were in estrus, 5 were in diestrus, and 1 was in proestrus; from the FEM 6-OHDA group, 3 females were in estrus, 4 were in diestrus, and 3 were in proestrus (Table 1).

**Table 1:** Above: Table showing the numbers of sham-operated (SHAM) and 6-hydroxydopamine (6-OHDA) lesioned female rats identified by vaginal cytology as being in estrus (EST), diestrus (DI), or proestrus (PRO) on the day that SHAM or 6-OHDA lesions were made (Day of Surgery) and on the day of testing for the What-Where-When Episodic-like memory task (Day of Testing).

**Females**	**EST**	**DI**	**PRO**
**SHAM**			
Day of lesion surgery	3	6	2
Day of testing	5	5	1
**6-OHDA**			
Day of lesion surgery	3	5	2
Day of testing	3	4	3
**LESION SIZE**			
6-OHDA lesion size for females in estrus (n = 3), diestrus (n = 5), or proestrus (n = 2) on the day of surgery	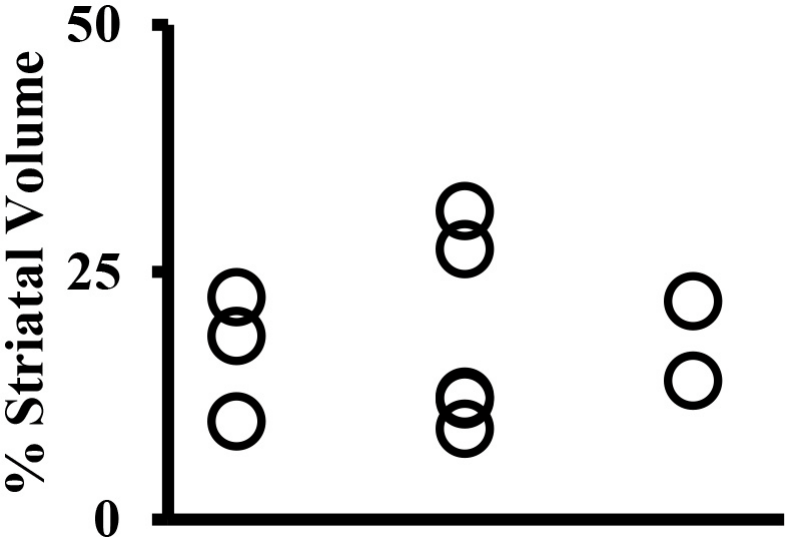		

*Below: Scatter plots showing the volumes of 6-OHDA lesions, expressed as percent of total caudate nucleus volume, for female rats that were identified as being in EST, DI, or PRO on the day of surgery*.

The efficacies of GDX and of GDX with hormone replacements were verified in expected group differences in the weights of the androgen-sensitive bulbospongiosis muscles (BSM). Specifically, average BSM weights were larger in the gonadally intact groups (MALE SHAM, MALE-6-OHDA) and in the GDX+TP 6-OHDA rats compared to muscle mass in the GDX 6-OHDA and GDX+E 6-OHDA groups (Table 2). One-way ANOVAs confirmed that there were significant main effects of Group for BSM mass [*F*_(4, 41)_ = 94.634, *p* < 0.0001]. Follow-up *post-hoc* comparisons further confirmed that BSM weights in GDX 6-OHDA and GDX+E 6-OHDA rats were similar to each other and were significantly lower than BSM mass in the gonadally intact (MALE SHAM, MALE 6-OHDA) and GDX+TP 6-OHDA groups (all *p* < 0.0001).

**Table 2:** Group mean weights of the androgen-sensitive bulbospongiosis (BSM) mass [in grams ± standard error of the mean (SEM)] for gonadally intact sham-operated (SHAM) and 6-hydroxydopamine lesioned (6-OHDA) males and for the males that were gonadectomized (GDX), GDX and supplemented with testosterone propionate, or GDX supplemented with 17β-estradiol and 6-OHDA lesioned (GDX 6-OHDA, GDX+TP 6-OHDA, GDX+E 6-OHDA, respectively).

**MALES**	**MEAN BSM (g) ± SEM**
SHAM	1.67 ± 0.05
6-OHDA	1.64 ± 0.05
GDX 6-OHDA	0.53 ± 0.03******
GDX+TP 6-OHDA	0.96 ± 0.08
GDX+E 6-OHDA	0.51 ± 0.03******

One-way analyses of variance identified significant main effects of Group on BSM weight; the allowed post-hoc comparisons confirmed that BSM weights of the GDX 6-OHDA and GDX+E 6-OHDA males were similar to each other and were significantly different (lower) than muscle weights for the gonadally intact MALE SHAM (*), gonadally intact MALE 6-OHDA (**), and GDX+TP 6-OHDA groups (***) (all p <0.0001).

### 6-OHDA and SHAM Lesions

**Figure 2:**
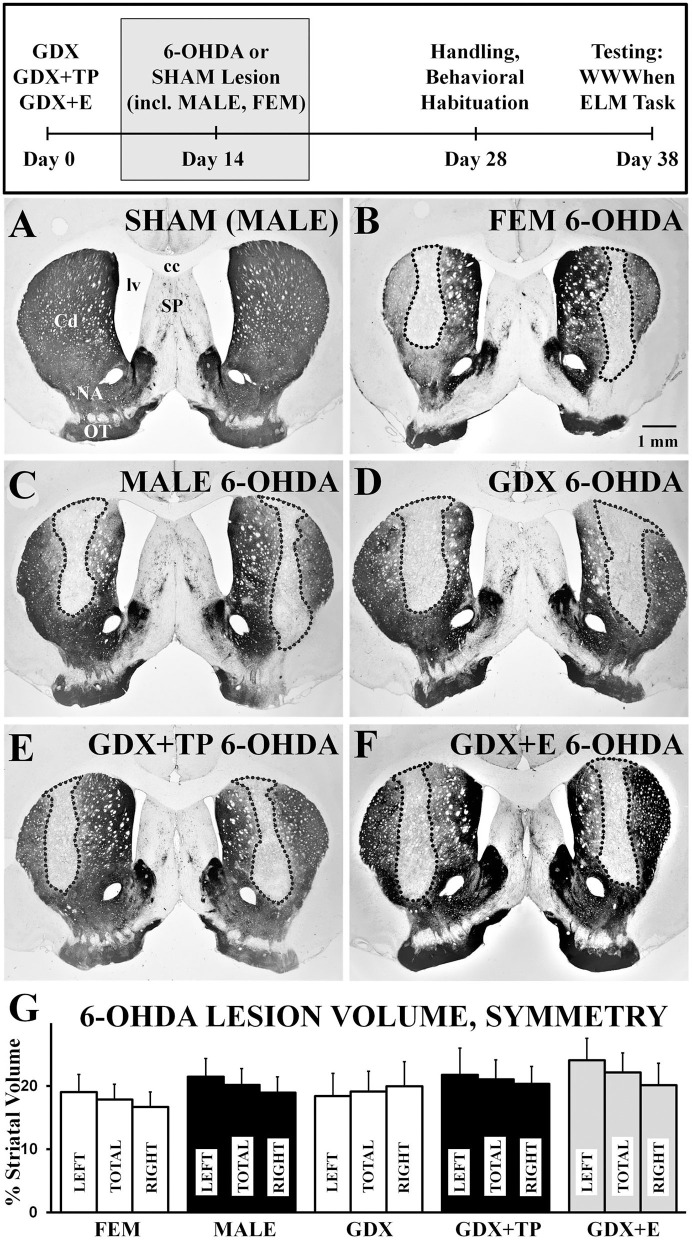
Representative low-power, brightfield light microscopic images of coronal sections through septal levels of the caudate nucleus (Cd) that are immunoreacted for tyrosine-hydroxylase (TH) **(A–G)**. The section from a gonadally intact sham-operated male (**A**, SHAM) shows dense labeling throughout the caudate. Sections from a 6-hydroxydopamine (6-OHDA) lesioned female (**B**, FEM 6-OHDA), a 6-OHDA lesioned male (**C**, MALE 6-OHDA) and from 6-OHDA lesioned rats that were gonadectomized (**D**, GDX 6-OHDA), GDX and supplemented with testosterone propionate (**E**, GDX+TP 6-OHDA), or GDX and supplemented with 17β-estradiol (**F**, GDX+E 6-OHDA) all show discrete bilateral zones of diminished TH-immunostaining (outlined). The experimental timeline showing when lesions and sham lesion were made is shown above, and bar graphs **(G)** and bar graphs showing group average sizes and symmetries of 6-OHDA lesions, expressed as percentages of the left (LEFT), right (RIGHT), and total (TOTAL) caudate nucleus volumes ± standard error of the mean, are shown below for all groups below. cc, corpus callosum; NA, nucleus accumbens; SP, septal nucleus; lv, lateral ventricle; OT, olfactory tubercle. Scale bar in **B** = 1 mm.

Light microscopic evaluations of TH-immunoreacted coronal sections showed that SHAM lesions had no discernible effects on staining intensity or striatal integrity in either male or female rats (Figure 2A). In contrast, injections of 6-OHDA produced discrete, bilateral zones of markedly diminished TH-immunostaining in all lesioned groups (Figures 2B–F). These sites were cylindrically shaped, about 0.5–1 mm in diameter, and were centered on the middle third of the caudate at roughly mid-septal levels. These sites also tended to be symmetrical (Figures 2B–F) and, on average, occupied 17–24% of the left, right, and total caudate nucleus (neostriatal) volumes in all groups (Figure 2G). There were, however, a small number of subjects in each group where left/right hemispheric differences in lesion volume exceeded 20%. There rats were all carefully assessed for evidence of circling or turning bias during WWWhen testing; this identified 2 rats (1 FEM 6-OHDA, 1 GDX 6-OHDA) where strong left-right turning biases and tight circling were noted, leading both to be removed from the study. Among the remaining subjects, statistical comparisons (one-way ANOVAs) confirmed that there were no significant or near significant main effects of Group on measures of lesion volume or lesion symmetry for either gonadally intact females vs. males or among the four male groups. Further, although not statistically assessed, there were no observed differences in lesion volume for females that were in estrus, diestrus, or proestrus on the day of lesion surgery (Table 1). Regression analyses that compared BSM weights to 6-OHDA-induced striatal lesion sizes in males were also found to be mainly non-significant (*R*^2^ = 0.04–0.19); the only significant relationship identified between BSM weight and lesion size was in the MALE 6-OHDA group (*R*^2^ = 0.752, *p* = 0.011), where the BSM dataset was incomplete.

### What-Where-When Performance

#### Non-object Exploration (Other) Behaviors (S1, S2, TT)

**Figure 3:**
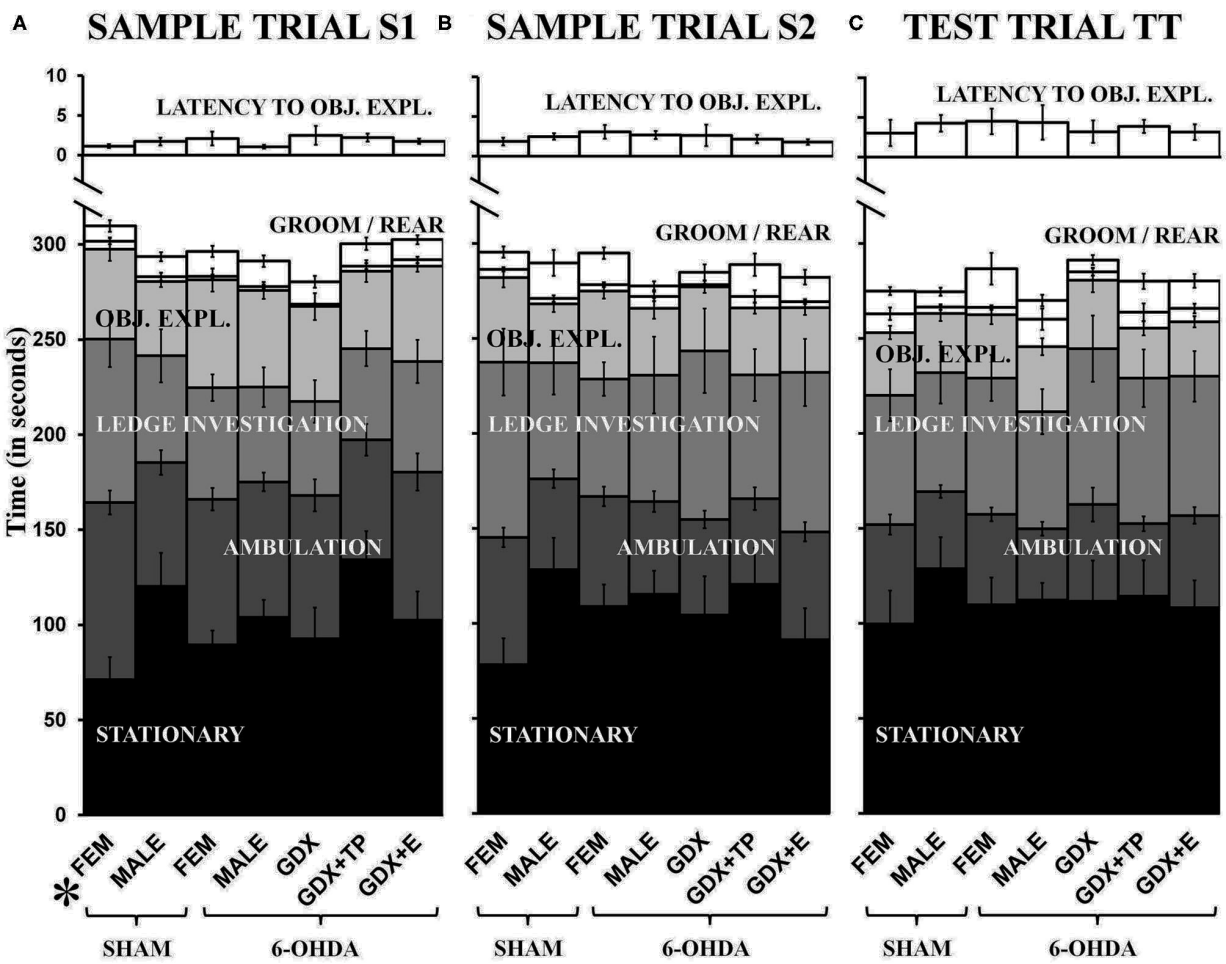
Stacked bar graphs showing average amounts of times in seconds, ± standard error of the mean, that rats spent stationary (black), ambulating (dark gray), engaged in ledge investigation (gray), object exploration (OBJ. EXPL., light gray), and grooming or rearing (GROOM/REAR) (white) during sample trial S1 **(A)**, sample trial S2 **(B)**, and the test trial TT **(C)**. The average latency (in seconds) from trial start to exploration of a first object (LATENCY TO OBJ.EXPL.) is also shown in white above on different time scale. Comparisons from left to right show that with the exceptions of higher levels of S1 ambulation and lower levels of S1 stationary behavior in gonadally intact sham lesioned females (SHAM, FEM), rats in all other groups [gonadally intact, sham lesioned males (SHAM, MALE), MALE and FEM rats that were 6-hydroxydopamine-lesioned (6-OHDA), and 6-OHDA male rats that were gonadectomized (GDX), GDX and supplemented with testosterone propionate (GDX+TP), or GDX and supplemented with 17β-estradiol (GDX+E)] apportioned trial times similarly. The *post-hoc* comparisons performed after the identification of significant main effects of Group and significant interactions between Behavior and Group for MALE/FEMALE comparisons for S1 showed that these effects were driven by significantly increased ambulation and decreased stationary behavior in SHAM operated FEM compared to SHAM operated MALES (*p* = 0.002, 0.012).

Latency to explore objects and times spent grooming, rearing, investigating the arena ledge, ambulating, and remaining stationary were all assessed during both sample trials (S1, S2) and during the test trial (TT). These data showed that rats in most groups spent similar amounts of trial times engaged in these discrete activities, and showed similar systematic increases or decreases in certain behaviors across trials (Figure 3). Specifically, rats in all groups initiated exploration of objects within about 1 second during S1 and by TT were waiting for closer to 5 s to begin interacting with objects (Figure 3, top panel). Rats in all groups also spent minimal times grooming (6–20 s) and small but gradually increasing amounts of time rearing from S1 (1–4 s, Figure 3A) to TT (4–15 s, Figure 3C). More time was spent investigating the arena ledge, ambulating, or remaining stationary. However, while rats in most groups were stationary for roughly 90–120 s of all trial times, during S1 (but not other trials, Figure 3C) the FEM SHAM were stationary for on average <60 s (Figure 3A). Conversely, for ambulation, rats in most groups engaged in locomotion for more than 60 s during S1 (Figure 3A), but by the TT only engaged in locomotion for about 45 s (Figure 3C). In contrast, the FEM SHAM group initially spent 90 s or more ambulating during S1 (Figure 3A) before decreasing locomotion to scores that were similar to the other groups in S2 and TT (Figures 3B,C). Similarly, for ledge investigation, most groups spent 60 s or less of trial times (S1, S2, TT) at the arena edge (Figure 3). However, the FEM SHAM group again engaged in more ledge investigation ~90 s, during S1 and S2 (Figures 3A,B). By the TT, however, all groups were spending roughly 60 s in investigating the arena's ledge (Figure 3C). Within-trials repeated-measures ANOVAs confirmed that there were significant main effects of Behavior for all trials and for both sets of group contrasts [gonadally intact females vs. males: *F*_(1.47−1.60, 57.32−62.23)_ = 71.14–103.37, *p* < 0.0001; all male groups: *F*_(1.39−1.59, 67.86−77.91)_ = 73.74–119.38, *p* < 0.0001]. Significant main effects of Group and significant interactions between Behavior and Group were also identified for S1 for in the gonadally intact female vs. male contrast [Group: *F*_(3, 39)_ = 3.04, *p* = 0.04; Behavior x Group: *F*_(4.78, 62.13)_ = 2.50, *p* = 0.042]; allowed *post-hoc* comparisons confirmed that these main effects were driven by significantly higher levels of ambulation and significantly lower levels of stationary behaviors in the FEM SHAM compared to MALE SHAM group during the S1 trial (*p* = 0.002 and 0.007, respectively). Repeated-measures ANOVAs that separately compared behaviors across trials and groups identified significant main effects of Trial for latency to explore objects, rearing, ambulation, and stationary behavior in the gonadally intact female vs. male groups contrast [*F*_(1.40−2.00, 54.42−78.00)_ = 3.57–71.84, *p* = 0.000–0.007] and for latency to explore objects, rearing, ambulation, and ledge investigation among the male groups [*F*_(1.41−2.00, 69.25−98.00)_ = 7.06–49.02, *p* = 0.000–0.005]; no significant or near significant main effects of Group and no significant or near significant interactions between Group and Trial were identified for either groups contrast. Finally, regression analyses that assessed within groups differences in discrete Other Behaviors as functions of 6-OHDA lesion sizes were overwhelmingly non-significant (*R*^2^ = 0.00–0.34). Across all behaviors, trials, and groups, only two significant relationships were identified. These were positive correlations in the GDX 6-OHDA group between 6-OHDA lesion size and stationary behavior during S2, and between lesion size and latency to explore objects during TT (*R*^2^ = 0.41, *p* = 0.047, and *R*^2^ = 0.45, *p* = 0.039, respectively).

#### Object Exploration: Total Object Exploration (S1, S2, TT)

Rats in all groups spent similar total amounts of time exploring objects within trials and progressively less time exploring objects from S1 to S2 to TT trials (Figure 3). Specifically, all cohorts explored objects for, on average, nearly 60 s during S1 (Figure 3A), for 30–45 s in S2 (Figure 3B), and for 25–35 s in TT (Figure 3C). Separate one-way ANOVAs that compared these times within trials for the two contrasts (gonadally intact female vs. male groups and among all male groups) found no significant or near significant main effects of Group for Total Object Exploration for any trial. Across-trials comparisons (repeated-measures ANOVAs) for groups contrasts further identified significant main effects of Trial [gonadally intact female vs. male groups: *F*_(2, 78)_ = 21.42, *p* < 0.0001; all male groups: *F*_(1.90, 92.86)_ = 35.19, *p* < 0.0001] but no significant or near significant main effects of Group and no significant or near significant interactions between Trial and Group for either comparison. Regression analyses that assessed total object exploration as a function of lesion size in the 6-OHDA groups were predominantly non-significant (*R*^2^ = 0.000–0.25). However, a significant positive relationship between lesion size and total object exploration was found for the GDX+E cohort for the TT (*R*^2^ = 0.49, *p* = 0.017). Finally, comparisons were also made in which the data were stratified by the order in which quadruplicate object sets were presented (Object Order). These within-groups, across-trials repeated-measures ANOVAs found no significant or near significant main effects of Object Order and no significant or near significant interactions between Object Order and Trial for total object exploration for any group.

#### Object Exploration: Individual Object Exploration (S1, S2)

Analyses of rats' investigations of individual objects showed that in both of the sample trials (S1, S2) rats in all groups divided exploration times more or less evenly and spent ~10–20 s exploring each object in S1 (Table 3A) and 5–15 s exploring each object in S2 (Table 3B). Within-groups repeated-measures ANOVAs identified isolated cases where main effects of Individual Object were significant: for FEM SHAM [*F*_(2.50, 24.98)_ = 12.45, *p* < 0.0001] and GDX 6-OHDA [*F*_(3, 27)_ = 5.53, *p* = 0.004] in S1; for GDX 6-OHDA in S2 [*F*_(3, 27)_ = 4.50, *p* = 0.011]. The objects and exploration times that drove these main effects were identified in follow-up pair-wise comparisons and are shown in bold in gray-shaded cells in Tables 3A,B.

**Table 3:** Average times (in seconds) of exploration of individual objects during sample trial S1 **(A**) and sample trial S2 **(B)**.

**Object**	**Key**	**FEM SHAM**	**FEM 6-OHDA**	**MALE SHAM**	**MALE 6-OHDA**	**GDX 6-OHDA**	**GDX+TP 6-OHDA**	**GDX+E 6-OHDA**
**(A) SAMPLE TRIAL S1: Total Individual Object Exploration (±SEM)**								
**Duration of Exploration Bout (±SEM)**								
**Object** **1**	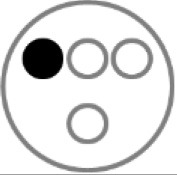	7.25 ± 1.35 0.90 ± 0.16	13.69 ± 4.27 1.33 ± 0.30	8.58 ± 1.51 0.99 ± 0.15	10.61 ± 1.78 0.84 ± 0.07	14.43 ± 2.46 1.07 ± 0.08	10.50 ± 2.38 1.09 ± 0.12	11.60 ± 2.13 1.06 ± 0.14
								
**Object** **2**	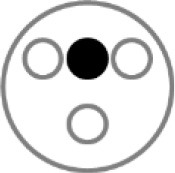	9.95 ± 1.67 1.06 ± 0.12	14.52 ± 1.95 0.87 ± 0.10	11.42 ± 1.09 1.03 ± 0.19	13.05 ± 1.86 0.76 ± 0.08	11.13 ± 1.25 0.86 ± 0.05	14.07 ± 3.37 0.93 ± 0.12	15.68 ± 2.29 0.91 ± 0.05
								
**Object** **3**	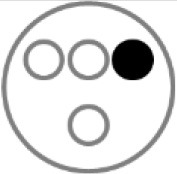	10.05 ± 2.30 1.09 ± 0.11	12.93 ± 2.65 1.01 ± 0.11	9.26 ± 1.76 1.07 ± 0.11	10.69 ± 1.99 0.91 ± 0.11	**8.42 ± 1.80** 0.98 ± 0.11	7.64 ± 0.87 1.10 ± 0.11	10.79 ± 0.99 1.00 ± 0.09
								
**Object** **4**	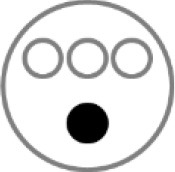	**20.08 ± 2.66** 1.22 ± 0.11	15.45 ± 3.42 0.99 ± 0.13	9.76 ± 1.35 1.14 ± 0.09	16.49 ± 3.13 **1.16 ± 0.12**	**18.48 ± 2.83** 1.14 ± 0.12	12.03 ± 3.11 0.96 ± 0.07	12.13 ± 2.04 0.91 ± 0.09
**(B) SAMPLE TRIAL S2: Total Individual Object Exploration (±SEM)**								
**Duration of Exploration Bout (±SEM)**								
**Object** **1**	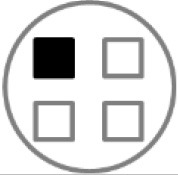	8.06 ± 1.77 1.06 ± 0.15	11.28 ± 2.35 1.05 ± 0.14	9.53 ± 2.70 0.88 ± 0.11	7.86 ± 1.86 0.95 ± 0.10	7.25 ± 1.56 0.89 ± 0.11	7.90 ± 1.73 0.93 ± 0.11	5.90 ± 1.44 0.93 ± 0.13
								
**Object** **2**	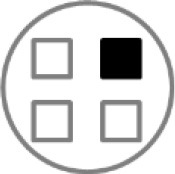	8.96 ± 1.64 1.17 ± 0.18	12.83 ± 2.17 1.20 ± 0.20	5.39 ± 1.27 0.95 ± 0.18	5.53 ± 1.45 0.74 ± 0.11	**4.59 ± 1.16** 0.97 ± 0.13	9.55 ± 3.38 1.25 ± 0.15	8.91 ± 2.65 1.07 ± 0.19
								
**Object** **3**	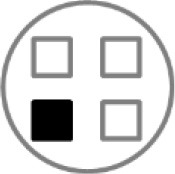	13.63 ± 2.12 1.08 ± 0.08	10.68 ± 2.00 1.10 ± 0.13	8.48 ± 1.07 0.98 ± 0.09	10.61 ± 1.98 0.91 ± 0.22	9.78 ± 1.49 0.97 ± 0.08	9.93 ± 2.08 0.88 ± 0.12	7.75 ± 1.63 0.77 ± 0.13
								
**Object** **4**	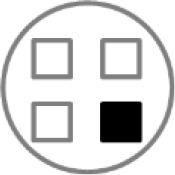	13.77 ± 2.63 1.16 ± 0.12	11.44 ± 2.07 1.10 ± 0.13	7.65 ± 2.72 1.27 ± 0.09	11.21 ± 3.25 1.03 ± 0.15	12.10 ± 1.91 1.37 ± 0.23	7.82 ± 1.49 1.08 ± 0.15	11.48 ± 1.56 1.05 ± 0.07

*Schematics shown on the left show the relative position/identity of the individual object assessed; the values located in the adjacent cells show average total exploration times [in sec, ± standard error of the mean (SEM)] (top) and average durations of separate bouts of exploration (±SEM) (bottom) for that object for gonadally intact sham-operated females (FEM SHAM) and males (MALE SHAM), for gonadally intact 6-hydroxydopamine lesioned (6-OHDA) females (FEM 6-OHDA) and males (MALE 6-OHDA), and for 6-OHDA male rats that were gonadectomized (GDX), GDX and supplemented with testosterone propionate, or GDX and supplemented with 17β-estradiol (GDX 6-OHDA, GDX+TP 6-OHDA, and GDX+E 6-OHDA). Within-groups comparisons (repeated-measures analyses of variance) of total times spent exploring individual objects identified significant main effects of Object for FEM SHAM and GDX 6-OHDA groups in S1 and for GDX 6-OHDA rats in S2. Significant main effects of Object for measures of durations of individual bouts of object exploration were also found for the MALE 6-OHDA group in S1. The individual objects driving these main effects identified in follow-up pair-wise post-hoc comparisons are shown in bold, in gray-shaded cells*.

The durations of individual instances or bouts of object exploration were also evaluated (Table 3). This measure was similar and similarly brief for all groups (<2 s) in both sample trials (Table 3). Within-groups comparisons (repeated-measures ANOVAs) generally found that main effects of Object Exploration Duration were non-significant. However, a significant main effect of Object Exploration Duration was identified for the MALE 6-OHDA group, albeit only for S1 (*F*_(3, 30)_ = 5.50, *p* = 0.004). Follow-up pair-wise comparisons identified the object-specific measure of exploration duration that drove this main effect, which is shown in bold in a gray-shaded cell in Table 3A. Finally, regression analyses showed that 6-OHDA lesion size was most often not a significant predictor of either mean exploration times for individual objects (*R*^2^ = 0.000–0.253) or mean durations of individual bouts of object exploration (*R*^2^ = 0.000–0.38) during S1 or S2. The single exception was a significant positive relationship identified for GDX+TP group between larger lesion size and longer mean exploration times for individual objects during S2 (*R*^2^ = 0.42, *p* = 0.031).

#### Object Exploration: Discrimination Indices (TT)

During test trials, rats' observation times were unevenly distributed among the 2 “old familiar” (OF) and 2 “recent familiar” (RF) objects present. Discrimination Indices (DI) calculated from these differences were compared across groups using repeated-measures ANOVAs. These identified: significant main effects of Group for comparisons of gonadally intact female and male groups [*F*_(3, 39)_ = 19.51, *p* < 0.0001] and for comparisons of all male groups: [*F*_(4, 49)_ = 9.31, *p* < 0.0001]; significant main effects of Discrimination among the males [*F*_(1.22, 59.99)_ = 14.07, *p* < 0.001]; and a significant interaction between Discrimination and Group for the gonadally intact female vs. male comparisons [*F*_(3.67, 47.70)_ = 3.04, *p* = 0.003]. The allowed *post-hoc* comparisons (Bonferroni-corrected pair-wise comparisons) are presented along with additional analyses separately below for discriminations of “What,” “Where,” and “When.”

##### What' discrimination

**Figure 4:**
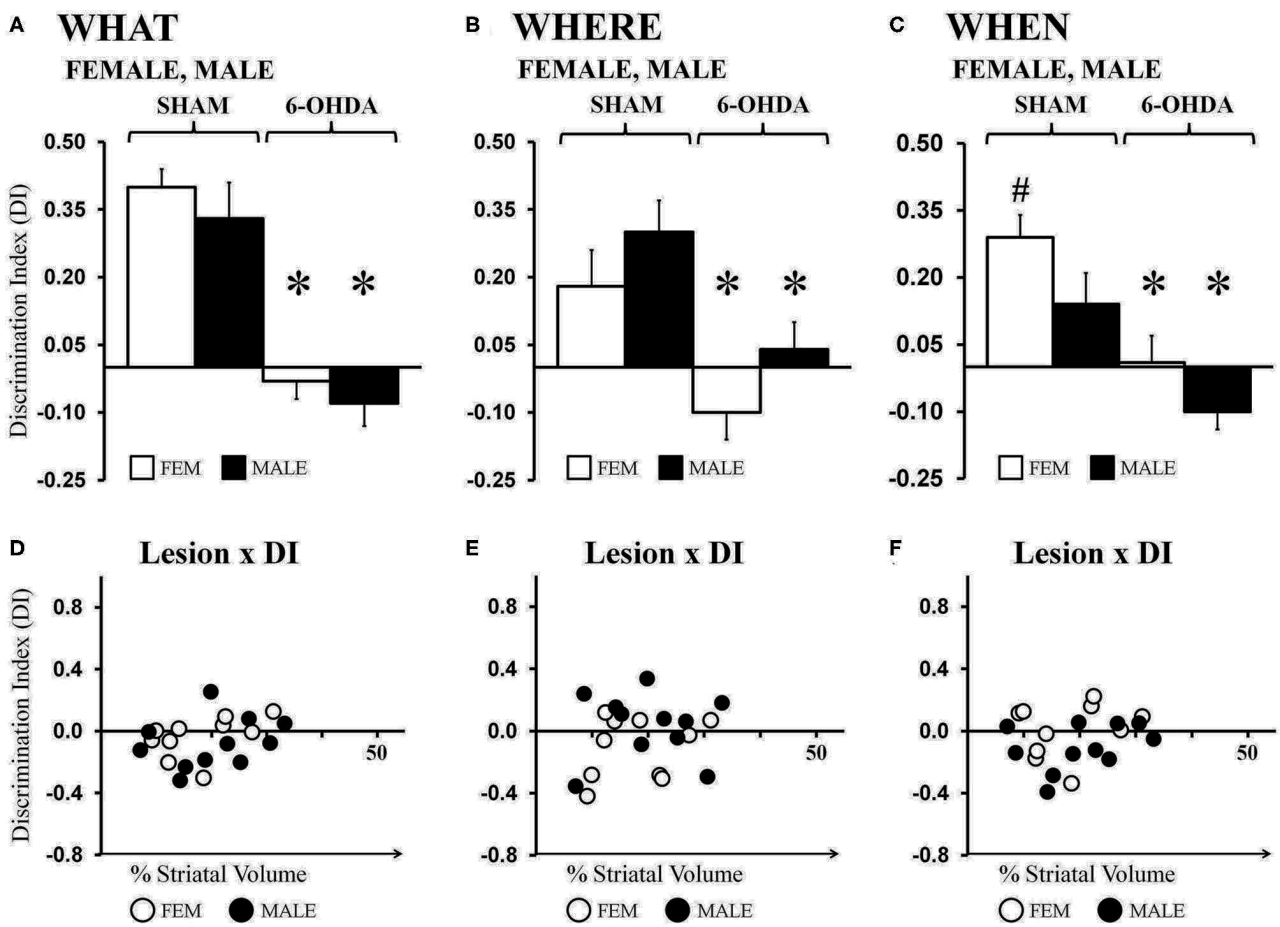
Bar graphs showing average “What” **(A)**, “Where” **(B)**, and “When” **(C)** discrimination indices (DIs) calculated from object exploration in Test Trials for gonadally intact sham-operated (SHAM) and 6-hydroxydopamine lesioned (6-OHDA) female (FEM, white) and male (MALE, black) rats. DIs for all domains were robust for FEM SHAM and MALE SHAM; trends for MALE over FEM advantage for “Where,” and FEM over MALE advantage for “When” were also seen. In contrast, DIs for all domains were impaired in FEM 6-OHDA and MALE 6-OHDA groups. Results from *post-hoc* comparisons are shown as follows: significant differences in DIs between the SHAM and 6-OHDA lesioned female and male rats (*); near significant differences in “When” discrimination between the SHAM-lesioned FEM and MALE groups (*p* = 0.053, #). Scatter plots of “What” **(D)**, “Where” **(E)**, and “When” **(F)** DIs expressed as functions of the size (percent of total striatal volume) of 6-OHDA lesions for individual gonadally intact FEM (white circles) and MALE (black circles) rats show no significant or consistent relationships between the two.

Average “What” DIs calculated for gonadally intact FEM SHAM and MALE SHAM rats were 0.40 and 0.33, respectively. Their preferential investigation of “old familiar” (S1) vs. “recent familiar” (S2) objects contrasted sharply with the average DIs calculated for FEM 6-OHDA and MALE-6-OHDA groups, which were −0.03 and −0.08, respectively (Figure 4A). *Post-hoc* comparisons (Bonferroni corrected alpha 0.0491) confirmed that “What” DIs were similar for the FEM SHAM and MALE SHAM groups; were similar for FEM 6-OHDA and MALE 6-OHDA groups; but were significantly lower for MALE 6-OHDA and FEM 6-OHDA rats vs. MALE SHAM and FEM SHAM groups (all *p* < 0.0001). Group differences in the robustness of discrimination were further supported in one-sample *t*-tests showing that “What” DIs were significantly >0 for FEM SHAM [*t*_(1, 10)_ = 9.55, *p* < 0.0001] and MALE SHAM rats [*t*_(1, 10)_ = 4.23, *p* = 0.002], but were not significantly different from zero for the FEM-6-OHDA or MALE 6-OHDA groups. Finally, regression analyses showed that for both sexes, the negative impacts of 6-OHDA lesions on “What” discrimination were not significantly predicted by individual differences in lesion size (FEM 6-OHDA: *R*^2^ =0.20; MALE 6-OHDA: *R*^2^ = 0.08, see Figure 4D).

**Figure 5:**
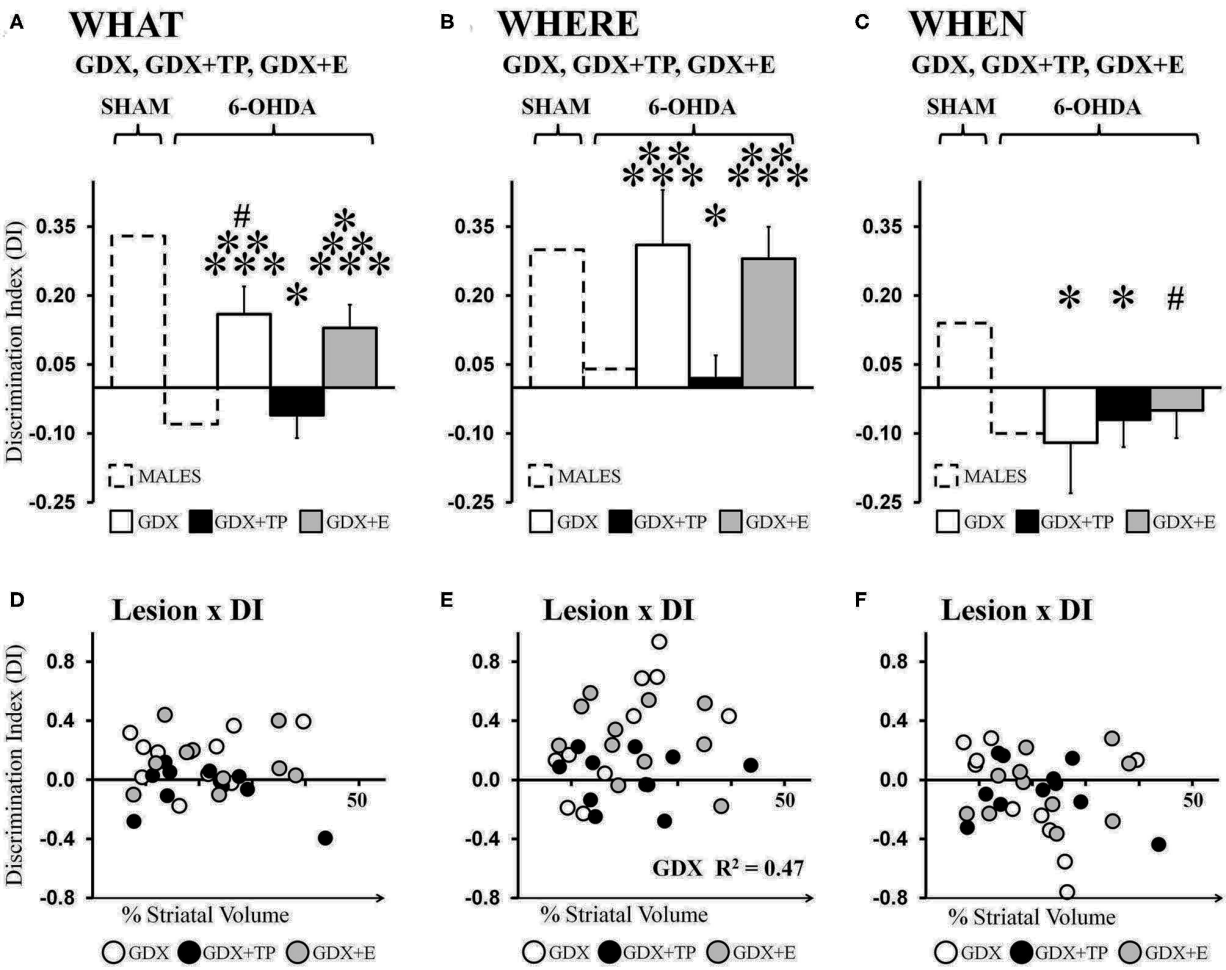
Bar graphs showing average “What” **(A)**, “Where” **(B)**, and “When” **(C)** discrimination indices (DIs) calculated from object exploration in Test Trials for 6-hydroxydopamine (6-OHDA) lesioned male rats that were gonadectomized (GDX, white), that were GDX and supplemented with testosterone propionate (black), or GDX and supplemented with 17β-estradiol 6-OHDA (gray); data from gonadally intact SHAM and 6-OHDA lesioned MALES are shown for reference (dashed outline). The GDX+TP cohort was profoundly impaired for “What” **(A)**, “Where” **(B)**, and “When”**(C)** discriminations. In contrast, DI's for the GDX and GDX+E groups were moderate for “What,” robust for “Where,” and poor for “When.” Results from allowed *post-hoc* comparisons of DI that included all of the male groups are shown as follows: significantly different than MALE SHAM (*); near significantly different than MALE SHAM (#); significantly different than MALE 6-OHDA (**); significantly different than GDX+TP 6-OHDA groups (***).Scatter plots below of “What” **(D)**, “Where” **(E)**, and “When” **(F)** DIs as functions of the size (percent of total striatal volume) of 6-OHDA lesions for individual GDX (white circles), GDX+TP (black circles), and GDX+E (gray circles) 6-OHDA rats show no consistent relationships between the two for “What” or “When” DI. For “Where” DI, a significant positive correlation was found between larger lesions and better “Where” DI for GDX 6-OHDA rats; the *R*^2^-value is shown on the plot **(E)**.

Analyses of data from the male groups showed that “What” DIs in GDX+TP 6-OHDA rats were low (−0.06) and were similar to those of the gonadally intact MALE 6-OHDA group. In contrast, DIs calculated for the GDX 6-OHDA and GDX+E 6-OHDA groups were 0.16 and 0.13, respectively. These values were lower than those observed for MALE SHAMs but higher than DIs calculated for the MALE 6-OHDA cohort (Figure 5A). *Post-hoc* comparisons (Bonferroni-corrected alpha = 0.0489) confirmed that “What” DIs in MALE 6-OHDA and GDX+TP 6-OHDA rats were similar to each other and one-sample *t*-tests showed that for both groups, these values were not significantly different from zero. Additionally, these analyses showed that “What” DIs for the GDX 6-OHDA and GDX+E 6-OHDA rats were similar to each other, but were significantly greater than DIs in the MALE 6-OHDA and GDX+TP 6-OHDA groups (*p* = 0.007–0.025), and significantly or near significantly lower than the “What” DIs of MALE SHAMs (GDX 6-OHDA: *p* = 0.05; GDX+E 6-OHDA: *p* = 0.02). One-sample *t*-tests showed that DIs for each group were significantly different from zero [GDX 6-OHDA: *t*_(1, 9)_ = 2.72, *p* = 0.024; GDX+E 6-OHDA: *t*_(1, 10)_ = 2.477, *p* = 0.033]. Regression analyses found no significant relationships between lesion sizes and the degrees of impairment or relative sparing observed for “What” discrimination for any of the 6-OHDA-lesioned groups [GDX 6-OHDA: *R*^2^ = 0.079; GDX+TP 6-OHDA: *R*^2^ = 0.163; GDX+E 6-OHDA: *R*^2^ = 0.002] (Figure 5D).

##### “Where” discrimination

Average “Where” DIs calculated for FEM SHAM and MALE SHAM rats were 0.18 and 0.30, respectively. In contrast, strong preference for displaced vs. stationary “old familiar” objects was not seen in the FEM 6-OHDA or MALE 6-OHDA groups; these two “Where” DIs were −0.10 and 0.04, respectively (Figure 4B). *Post-hoc* comparisons (Bonferroni-corrected alpha = 0.0491) showed that although the “Where” DI in the MALE SHAM group was noticeably greater than that of the FEMALE SHAM rats, and in the MALE-6-OHDA group compared to the FEM 6-OHDA subjects, these difference did not reach statistical significance. However, *post-hoc* testing did confirm that differences in DI's between the SHAM and 6-OHDA groups were significant for both sexes (females, *p* = 0.007; males, *p* = 0.009). One-sample *t*-tests further showed that “Where” DIs were significantly >0 for FEM SHAM [*t*_(1, 10)_ = 2.39, *p* = 0.038] and MALE SHAM [*t*_(1, 10)_ = 4.07, *p* = 0.002] rats but were not significantly different from zero for the FEM 6-OHDA or MALE 6-OHDA cohorts. Regression analyses also showed that for both sexes, the degrees to which “Where” discriminations were negatively impacted were not significantly predicted by differences in 6-OHDA lesion sizes (FEM 6-OHDA: *R*^2^ =0.07; MALE 6-OHDA: *R*^2^ = 0.00, see Figure 4E).

Average “Where” DIs calculated for 6-OHDA lesioned GDX, GDX+TP, and GDX+E rats were 0.16, −0.06, and 0.13, respectively (Figure 5B). These data indicate preferential investigation of the displaced vs. the stationary “old familiar” object in GDX 6-OHDA and GDX+E 6-OHDA groups but not in GDX+TP 6-OHDA rats. *Post-hoc* comparisons (Bonferroni-corrected alpha of 0.0489) confirmed that DIs were similar in MALE 6-OHDA and GDX+TP 6-OHDA rats; were similar in the MALE SHAM, GDX 6-OHDA, and GDX+E 6-OHDA groups; and were significantly different (lower) in MALE 6-OHDA and GDX+TP 6-OHDA rats compared to the MALE SHAM, GDX 6-OHDA, and GDX+E 6-OHDA groups (*p* = 0.013–0.032). Group differences in the robustness of “Where” DIs were also reflected in one-sample *t*-tests showing that, like MALE SHAMS, the DIs of GDX 6-OHDA, and GDX+E 6-OHDA rats were significantly >0 [GDX 6-OHDA: *t*_(1, 9)_ = 2.50, *p* = 0.034; GDX+E 6-OHDA: *t*_(1, 10)_ = 3.79, *p* = 0.004] but that, like MALE 6-OHDA rats, DIs from the GDX+TP 6-OHDA groups were not significantly different from zero. Lastly, regression analyses (Figure 5E) found little evidence for significant relationships between 6-OHDA lesion size and degrees to which “Where” DI was impaired (GDX+TP 6-OHDA: *R*^2^ = 0.00) or spared (GDX+E 6-OHDA: *R*^2^ = 0.09). The only significant relationship found was a positive correlation between larger lesion size and greater/better 'Where' discrimination in the GDX 6-OHDA group (*R*^2^ = 0.467, *p* = 0.029).

##### “When” discrimination

Average ‘When’ DIs calculated for FEM SHAM and MALE SHAM rats (0.29 and 0.14, respectively) indicated preferential investigation of the stationary “old familiar” object vs. the average investigation of the two “recent familiar” objects in both sexes that was consistently stronger in the FEM SHAM group. In contrast, average “When” DIs for FEM 6-OHDA and MALE 6-OHDA rats were similar to each other and much lower than those of sham-operated rats (0.01 and −0.10, respectively) (Figure 4C). *Post-hoc* comparisons (Bonferroni-corrected alpha of 0.0491) of “When” DIs identified the DIs of the FEM SHAM group as nearly significantly different (greater) than those of MALE SHAM rats (*p* = 0.053); the DIs of the FEM 6-OHDA and MALE 6-OHDA groups as similar to each other; and the DIs for the FEM SHAM vs. FEM 6-OHDA groups and for the MALE SHAM vs. MALE 6-OHDA groups as significantly different (*p* = 0.001, *p* = 0.003, respectively). One-sample *t*-tests also showed that “When” DIs: were significantly or near significantly >0 for FEM SHAM [*t*_(1, 10)_ = 5.49, *p* < 0.0001] and MALE SHAM [*t*_(1, 10)_ = 2.09, *p* = 0.064] groups; were not significantly different from zero for the FEM 6-OHDA group; and were significantly lower than zero for MALE 6-OHDA rats [*t*_(1, 10)_ = 2.33, *p* = 0.042]. Regression analyses also showed that 6-OHDA lesion size did not significantly predict the magnitude of “When” discrimination deficits in either sex (FEM 6-OHDA: *R*^2^ = 0.04; MALE 6-OHDA: *R*^2^ = 0.11, see Figure 4F).

Average “When” DIs calculated for GDX 6-OHDA, GDX+TP 6-OHDA, and GDX+E 6-OHDA rats (−0.12, −0.07, and −0.05, respectively) indicated poor discrimination in all three groups (Figure 5C). Allowed *post-hoc* comparisons (Bonferroni-corrected alpha = 0.0489) showed that “When” DIs in these groups were similar to each other, were similar to DIs in the MALE 6-OHDA group, but were significantly or nearly significantly different from DIs in the MALE SHAM group (GDX 6-OHDA: p = 0.016; GDX+TP 6-OHDA: *p* = 0.042; GDX+E 6-OHDA, *p* = 0.064). One-sample *t*-tests further showed that “When” DIs for GDX 6-OHDA, GDX+TP 6-OHDA, and GDX+E 6-OHDA groups were not significantly different from zero. Finally, regression analyses confirmed that “When” DI values were not significantly predicted by the size of 6-OHDA-induced striatal lesions in any of these groups (GDX 6-OHDA: *R*^2^ = 0.21; GDX+TP 6-OHDA: *R*^2^ = 0.10; GDX+E 6-OHDA: *R*^2^ = 0.06, see Figure 5F).

## Discussion

Memory impairments, including those involving episodic memory, present in roughly 20% of PD patients at or before the onset of motor deficits (30) and afflict more than 50% of patients over the course of illness (20, 26). Episodic memory impairments in particular can also predict a more rapid and more severe decline in motor and memory function (34, 35) and signal a greater probability of developing PD-related dementia (26, 36, 37). These clinical characteristics combine with the overall resistance of cognitive and mnemonic disturbances in PD to available therapeutics (19, 20, 22) to bring urgency to resolving questions about the neural underpinnings of episodic memory dysfunction in PD. While imaging studies have begun to identify structural brain changes that correlate with and in some cases predict the onset of episodic memory deficits in PD (42–44), less is known about the physiological factors that render episodic memory vulnerable in this disease. These factors could serve as biomarkers that prompt early, possibly preventive intervention and facilitate planning for long-term clinical care. These factors could also represent novel therapeutic targets that address unmet clinical needs for more effective treatment of the cognitive and mnemonic deficits in PD (19, 20, 22). This study was stimulated by findings suggesting that biological sex and/or sex hormones are among the factors that influence episodic memory function in PD (60–62) and used a preclinical PD model to investigate this further. Specifically, partial, bilateral neostriatal 6-OHDA dopamine lesions in female and male rats were paired with classical methods of hormone monitoring and manipulation and with behavioral testing using the WWWhen Episodic-Like Memory task. These studies confirmed and extended recent evidence for rats as suitable models for human sex differences in episodic memory (64). Thus, in keeping with sex differences described in human episodic memory for temporal and spatial information (71–79), non-significant trends were seen indicating that FEM SHAM rats outperformed MALE SHAM rats in “When” discrimination and gonadally intact that MALE SHAM rats tended to outperform FEM SHAM rats in “Where” discrimination. Further, these studies identified striking, negative impacts of 6-OHDA dopamine lesions on ELM function for all domains in gonadally intact rats of both sex. Finally, the data showed that ELM deficits in 6-OHDA lesioned male rats were strongly influenced by circulating hormone levels in domain specific ways. Specifically, similar to the gonadally intact males, 6-OHDA lesions in GDX+TP rats significantly impaired discriminations of “What,” “Where,” and “When.” However, in the 6-OHDA-lesioned GDX and GDX+E groups, “When” discrimination was fully impaired, “What” discrimination was partially disrupted, and “Where” discrimination remained fully intact. As discussed below, these patterns of memory impairment and sparing map to ELM domains recently identified as differentially sensitive to circulating estrogens and/or androgens (64) and offer a second, powerful example where the normally harmful effects of androgen depletion prove beneficial for memory function in 6-OHDA lesioned rats (66). First, however, the strategies used to minimize potential confounds from non-mnemonic influences on the ELM data are considered.

### Experimental Design and Data Analyses Minimize Non-mnemonic Confounds

Key variables examined in this study included biological sex and monitored and manipulated sex hormone levels. These variables require that study design and data interpretation be informed by known sex differences in key behaviors, and by the broad range of metabolic and motivational states that gonadal hormones modulate in sex-specific ways. These include prominent sex differences in and sex hormone impacts on, sensitivity to positive and negative reinforcement (80–88). Thus, while there are several options for laboratory testing of ELM in rodents (89–93), the WWWhen task was selected for its leveraging of rats' innate preference for novelty and spontaneous investigations of novel objects encountered in the environment (63, 92). In fact, this task shares many of the same benefits recently espoused for single trial object recognition tasks in investigating neuroendocrine influences on learning and memory (94). In addition to mitigating potential confounds associated with reward contingencies, the WWWhen task is also minimally stressful and thus reduces the potential for bias arising from sex-specific impacts of stress on cognition and affect in rats (95–97). Studies in rat showing that stress sex-specifically impacts dopamine physiology in brain regions including the neostriatum (98), and studies in humans showing negative impacts of stress on episodic memory performance (99–103) further reinforce the importance of adopting testing procedures in animal subjects that minimize this variable.

This study also included variables of partial, bilateral neostriatal 6-OHDA lesions. This brings additional possibility for sex- and sex hormone-specific caveats. For example, studies in rats and mice have shown that the extent of and/or susceptibility to the effects of toxin-induced dopamine lesions is greater in males than in females (104–107); is greater for females in diestrus compared to proestrus (108–110); is greater in gonadally intact compared to GDX males (104, 111); and is greater in GDX males supplemented with the non-aromatizable androgen, dihydrotestosterone, compared to GDX males treated with estradiol (104, 105, 112). Moreover, these differences have been shown to be especially to exclusively robust for moderately sized, partial 6-OHDA lesions (6, 104). For these reasons, rigorous quantitative evaluations were made in this study of 6-OHDA lesion volume and lesion symmetry in all subjects and groups. As in a previous study using a similar lesioning protocol (66), there was some inter-subject variance in lesion measures. However, these tended to be small. Further, there were no obvious correlations between lesion size and estrous cycle stage at the time of surgery among the female subjects; there were no significant or near significant correlations between lesion size and hormone status at the time of surgery in males; there were no significant or near significant group differences in 6-OHDA lesion size or symmetry; and other than a small number of isolated cases involving non-mnemonic behaviors, the only significant correlation found between lesion size and ELM was a single positive association between larger lesion size and improved “Where” discrimination in GDX 6-OHDA rats. This adds to arguments for the behavioral sparing in this group as being highly unlikely to be due to smaller lesion sizes.

Finally, this study used lesion strategies developed intentionally to model early stages of PD and to yield motor deficits that are minimal or absent (65, 113–117). Nonetheless, multiple behaviors were evaluated during both the Sample and Test trials in addition to object exploration/discrimination, to confirm that 6-OHDA lesioned rats had the same abilities to ambulate, navigate, and explore as SHAM lesioned controls. Importantly, there were no group differences among the 6-OHDA or SHAM groups in ambulation, rearing, grooming, arena ledge investigation, object exploration, or stationary behavior for any trial. Rather, the 6-OHDA and SHAM groups apportioned and modified their engagement in all major activities—including object exploration (below), similarly both within and across trials.

### ELM in a Preclinical Model of PD: Validation of Sham-Lesioned Controls

Partial unilateral or partial bilateral nigrostriatal dopamine lesions in rats and mice have been shown to elicit measurable changes in active avoidance, working memory, reference memory, object recognition, and/or other cognitive domains that model those that are at risk in early and pre-motor stages of PD (66, 115, 118–123). To our knowledge, however, the present study is the first to ask whether episodic memory deficits, which are also at risk in PD (21, 32, 33), are induced in a preclinical dopamine lesion PD model (partial bilateral neostriatal 6-OHDA lesions). As a critical control, we included cohorts of gonadally intact male and female rats that were sham lesioned, i.e., bilaterally injected with acidified vehicle. Evaluations of the injected neostriatal regions revealed no evidence of local tissue disruption or obvious change (increases or decreases) in the intensity of TH-immunostaining compared to adjacent, un-injected neostriatal zones. Nonetheless, both SHAM groups were systematically probed for possible effects on an array of task-related motor, exploratory, and other behaviors. These analyses revealed sex differences that were consistent with the increased activity/ambulation that has been reported for female compared to male rats in Novel Open Field testing (124, 125). Specifically, the FEM SHAM rats engaged in significantly more active behavior (ambulation, ledge investigation) and displayed significantly less inactivity (stationary behavior) than the MALE SHAM rats. Comparisons with published data from this lab, where WWWhen testing was carried out in un-operated, gonadally intact male and female rats (64), also showed similarities in both the proportions of trial times that SHAM vs. un-operated rats allotted to major task-related behaviors, and in calculated discrimination indices. Thus, for the latter DIs in SHAM and un-operated control male and female rats alike were some 1.2–2.4 times greater for “What” discrimination compared to discriminations of “Where” and “When” (64).

Sex differences in DIs observed for the MALE SHAM and FEM SHAM rats in this study were non-significant. However, trends in the data were observed that were similar to those identified in the two prior studies of ELM in rats that included biological sex as a covariate. For example, in keeping with findings for human males as outperforming females in tests of episodic memory requiring visuospatial recall (75, 76, 79), all rat studies including the present showed better “Where” discrimination in gonadally intact male compared to gonadally intact female subjects (64, 126). The present study also revealed a trend for better discrimination of “When” in FEM SHAM compared to MALE SHAM rats. This potentially aligns with findings showing that women perform better than men in temporal ordering tasks and in estimating temporal features of remembered events (71–74, 77, 78). However, despite the robust evidence that supports superior performance of women in tests of episodic memory requiring recall of pictures and objects (79, 127), no rodent studies to date– including the present, have identified corresponding significant or non-significant trends for female over male differences in rats' discriminations of “What.” While the presence or absence of sex differences could be related to the different cells and circuits that process what, where and when ELM domains [see (128)], they may also be explained by the likelihood that discriminations based on multiple object features (“What”) are more easily made than those based on more constrained dimensions of “Where” or “When” (94, 129, 130). Accordingly, it may be necessary to increase the mnemonic demands of the WWWhen task (e.g., lengthen inter-trial intervals or reduce the number of distinguishing dimensions for sample objects) in order to reveal the full extent to which human patterns of domain-specific sex differences in episodic memory are recapitulated in rats.

### ELM in a Preclinical Model of PD: Sex Differences

Sex differences characterize the incidence and prevalence of PD (10–13) and differentiate many of its motor, autonomic and affective disturbances (6, 12, 131–133). Although less intensively studied, consensus findings also link male gender to increased risk for developing PD-related cognitive dysfunction and dementia (5, 6, 58–60, 134). Episodic memory deficits have also been shown to be more common, and to worsen more rapidly in males (60) and to respond better to multimodal exercise interventions in females (61, 62). Thus, similar to what has been more firmly established for aging, Alzheimer's disease, and schizophrenia (50–55, 57), there are reasons to suspect that biological sex and sex hormones also influence episodic memory dysfunction in PD in potentially therapeutic ways. One objective of this study was to determine the utility of a preclinical, 6-OHDA lesion rat model of PD to more rigorously evaluate these influences. Using the WWWhen task and well-validated sham-lesioned controls (above), we showed that partial, bilateral neostriatal 6-OHDA lesions indeed induced significant, highly robust ELM deficits in both male and female rats. Specifically, rats of both sexes were profoundly impaired in discriminating among the Test trial objects based on domains of “What,” “Where,” and “When.” However, mindful of data from this lab and others showing that 6-OHDA lesions can induce potentially confounding perseveration, difficulty in disengaging from stimuli, and delays in initiating behavior (66, 135) we also evaluated 6-OHDA and SHAM rats for latency to initiate object exploration, total object exploration times, total amounts of time spent exploring individual objects, and mean durations of single bouts of object exploration across groups. Due perhaps to the small arena size and the proximity of objects to the central start position, there were no indications that 6-OHDA lesioned rats (of any group) hesitated at the starts of Sample or Test trials. Further, and possibly related to the relatively short distances that separated sample objects, there was no evidence that any of the 6-OHDA lesioned groups engaged in prolonged or perseverative explorations of individual objects. These findings reinforce conclusions that differential object investigations observed during Test trials aptly reflected rats' ELM and further identify the WWWhen task and the testing apparatus used as suitable for evaluating episodic memory dysfunction in 6-OHDA lesioned rats. However, in contrast to the need for increased mnemonic demands to resolve sex differences in ELM in gonadally intact animals, any sex differences that may have been present among the MALE 6-OHDA and FEM 6-OHDA rats were obscured by the extremely low levels of discrimination seen in both groups. Thus, shorter inter-trial intervals may be needed to avoid basement effects and determine whether a more severe PD-related memory phenotype in males that is predicted in the human literature (5, 6, 59, 60) is borne out in MALE 6-OHDA compared to FEM 6-OHDA rats. It may also be useful to evaluate ELM in 6-OHDA lesioned male and female rats sooner and/or at several intervals after the induction of chemical lesions. Although inarguably abrupt compared to the progression of dopamine loss in PD, the strategy used here of injecting 6-OHDA among the axon terminal fields of nigrostriatal DA neurons has been shown to produce a more protracted time course of dopamine depletion compared to 6-OHDA injections targeting the medial forebrain bundle or substantia nigra (113). Thus, while not without caveats, this model might be useful for exploring and better understanding the sex differences in onset and/or rates of memory decline that are seen in PD (5, 6, 59, 60). Genetic rodent models of PD such as PINK1 knockout rats, which have been shown to undergo progressive nigrostriatal dopamine loss (136, 137), may also be well- and perhaps better-suited for this purpose.

### ELM in a Preclinical Model of PD: Hormone Impacts in Males

We recently demonstrated the utility of pairing 6-OHDA lesions with classical hormone manipulations in male rats as important, previously untested means of modeling and exploring hormone impacts on cognitive dysfunction in PD. Specifically, we used Barnes maze testing to evaluate and compare spatial working memory, learning strategy, and other higher order functions in 6-OHDA-lesioned male rats that were either gonadally intact, gonadectomized (GDX), or GDX and supplemented with testosterone or estradiol (66). These analyses were informed by earlier work showing that the principal measures of Barnes maze performance assessed are impaired by GDX and are attenuated in GDX rats supplemented with testosterone but not estradiol i.e., are androgen-sensitive (138). What was found was that these androgen-sensitive elements of behavior (spatial working memory, other frontal lobe operations) were profoundly impaired by 6-OHDA lesions—but only in animal groups where circulating androgen levels were physiologic, i.e., only in male 6-OHDA lesioned gonadally intact and GDX+TP rats. These findings seem consistent with recent studies showing that the motor consequences of similar experimental dopamine lesions are also dependent on and/or exacerbated by circulating androgens in males (139, 140). However, we also found that in 6-OHDA lesioned GDX and GDX+E rats, Barnes maze performance rivaled that of un-lesioned, hormonally intact controls (66). Thus, in these two groups, neither the profound Barnes maze deficits that are normally induced by androgen depletion (138) nor those that are produced in control and GDX+TP by 6-OHDA lesions were present (66). As discussed below, data from the present study suggest that similarly intriguing and perhaps therapeutically relevant relationships also exist between experimental dopamine lesions, circulating androgens, and processes of ELM.

Recent studies from this lab carried out in un-lesioned rats showed that GDX in male rats profoundly impairs ELM for “What”, “Where”, and “When” discriminations (64). However, these studies also showed that GDX-induced deficits were rescued by estrogen and/or testosterone replacement in highly selective, domain specific ways (64). Specifically, GDX-induced deficits in “What” discrimination were fully attenuated by TP and were partially rescued by E, thus indicating a requirement for both estrogen and androgen signaling. In contrast, GDX-induced deficits in “Where” discrimination were fully attenuated by TP and were unaffected by E, thus indicating androgen-sensitivity and estrogen-insensitivity. Finally, GDX-induced deficits in “When” discrimination were fully and equally attenuated by both TP and E, indicating their estrogen-sensitivity and androgen-insensitivity (64). The present assessment of 6-OHDA lesion impacts on these same ELM domains, in these same groups (gonadally intact, GDX, GDX+E, GDX+TP), revealed profound lesion-induced deficits for “What,” “Where,” and “When” discrimination, albeit only in the MALE 6-OHDA and GDX+TP 6-OHDA groups. In contrast, in the GDX 6-OHDA and GDX+E 6-OHDA rats, there was selective sparing of 6-OHDA-induced deficits that was highly specific for androgen-sensitive discrimination domains. Thus, in both GDX 6-OHDA and GDX+E 6-OHDA rats, discrimination of “Where” was fully spared, discrimination of “What” was partially spared, and discrimination of “When” was fully vulnerable to 6-OHDA lesion-induced deficits. It is important to repeat that neither impairments nor sparing of ELM discriminations were related to differences in 6-OHDA lesion sizes. Rather, as previously seen for Barnes maze testing (66), the data were dependent on circulating androgen levels. Specifically, physiological levels of androgen, which normally support these and other cognitive functions (64, 81, 141–145), render these processes vulnerable to dysfunction induced by nigrostriatal dopamine depletion. The data also support the corollary that androgen depletion, which is normally harmful to cognition and memory (64, 138, 146, 147), protects these domains from dysregulation and dysfunction induced by experimental 6-OHDA lesions. Because the WWWhen task concurrently measures discriminations that are explicitly estrogen-sensitive (“When”), uniquely androgen-sensitive (“Where”), and requiring of both (“What”) (64), especially strong arguments can be made for androgens, and not estrogens, as conferring behavioral vulnerability and protection, and for the targeted behaviors as being androgen, and not estrogen-sensitive. One explanation for how this occurs could lie in off-setting impacts on prefrontal dopamine levels. Specifically, nigrostriatal 6-OHDA lesions have been shown to diminish dopamine levels in prefrontal and cingulate cortices (148, 149) whereas GDX has been shown to selectively increase dopamine levels in these cortical regions in an androgen-dependent, estrogen-insensitive manner (67, 150, 151). Thus, given the well-established inverted U-shaped function that defines the relationship between dopamine levels and prefrontal cortical functions (152), it is possible that the combination of neostriatal 6-OHDA lesions and GDX or GDX+E yield prefrontal dopamine levels that are more functionally optimal compared to rats that are 6-OHDA-lesioned (hypodopaminergic) or hormonally manipulated (hyperdopaminergic) alone. In MALE 6-OHDA and GDX+TP 6-OHDA rats, there are no hormone-related, dopamine-facilitating influences present to balance the prefrontal hypodopaminergia induced by 6-OHDA lesions, thus, predictably, leading to cognitive impairment (152–155). However, while prior studies linked androgen regulation/dysregulation to frontal lobe functions (66) the present studies extend this relationship to processes of ELM. This opens the possibility for androgen-mediated actions and mechanisms also targeting additional brain regions and neurotransmitters systems that along with the frontal lobe are critical for mediating this form of memory. These additional targets could include medial temporal lobe structures such as the entorhinal cortices, the hippocampal formation, and the septo-hippocampal cholinergic systems. All have been inexorably linked to episodic and other types of memory functions in healthy humans (156, 157), are strongly linked to the disturbances in episodic and other memory processes in PD (44, 158, 159) and are highly sensitive to androgens in rats (160–162). Data from rodent studies further underscore the need to evaluate and compare androgen impacts on lateral and medial entorhinal cortices and on CA1 and CA3 hippocampal subfields. Like gonadal hormones, these loci have been shown to play striking, domain-specific roles in what, where, and where elements of episodic memory [e.g., (128, 163–169)]. Finally, there are intriguing data showing pivotal roles for hippocampal area CA3 (89) and for functional interactions between medial prefrontal cortex and the CA1 and CA3 subfields (170–172) in the integration of “What,” “Where,” and/or “When” information into cohesive episodic memories. Identifying the hormone sensitivities of these important sites, circuits, and mnemonic processes, however, will require studies that not only combine hormone manipulations with site specific and disconnection lesion strategies but that also use an alternate version of the WWWhen task that incorporates recent and old familiar objects that are displaced into the test trial (173). Unlike the version of the WWWhen task used here, the test trial configuration of this task allows for explicit measurement of interactions between memory for object location and temporal ordering (128). While data from this lab have shown that hormone modulation of memory functions tapped in the version of the WWWhen task used here (64) differs from hormone sensitivities identified for memory processes engaged in preference tasks based on object features (174) or location (175) alone, testing on the alternative WWWhen task will be important in more definitively tying hormone impacts to the integrative aspects of ELM.

## Conclusions and Future Directions

Both the motor signs and the non-motor symptoms of PD are distinguished by prominent sex differences (5, 7–9, 12, 58, 59). This study asked how gonadal hormones influence the cognitive impairments of PD in hopes of discovering new ways to more effectively combat these largely treatment-resistant aspects of the disease. Focusing specifically on processes of episodic memory, we found that this memory form is highly susceptible to impairment caused by a 6-OHDA dopamine lesion model of PD in male and female rats. However, in male rats, we also found that 6-OHDA-induced impairments in discrimination domains previously identified as androgen-sensitive (64) could be strongly attenuated by reducing circulating androgen levels. Understanding the basis for these potentially therapeutic actions may be especially pressing to resolve, given the prevalence of low testosterone levels in PD patients (176) and current practices of using hormone replacement therapies to elevate them (177). With the present identification of 6-OHDA lesions and WWWhen ELM testing in rats as a validated experimental framework, suitable means are now at hand for deeply investigating these actions and uncovering their neurobiological bases.

## Data Availability Statement

All datasets generated for this study are included in the article/Supplementary Material.

## Ethics Statement

The animal study was reviewed and approved by Institutional Animal Care and Use Committee at Stony Brook University.

## Author Contributions

MC is a Ph.D. candidate who helped develop the experiments and was responsible for behavioral testing, for data analysis and archiving, for figure preparation, and for contributing to manuscript writing and editing. DJ is an undergraduate student who assisted with animal handling, behavioral analyses, data archiving, and manuscript editing. BA is a Professor in Stony Brook University's Department of Psychology who assisted in developing the experiments, provided guidance for behavioral testing and analysis, and contributed to manuscript editing. MK is a Professor in Stony Brook University's Department of Neurobiology and Behavior who developed the experiments, provided oversight for all aspects of the study, assisted with behavioral testing and histology, and contributed to manuscript writing and editing. All authors contributed to the article and approved the submitted version.

## Conflict of Interest

The authors declare that the research was conducted in the absence of any commercial or financial relationships that could be construed as a potential conflict of interest.
